# Body mass index at age 18–20 and later risk of spontaneous abortion in the Health Examinees Study (HEXA)

**DOI:** 10.1186/s12884-015-0665-2

**Published:** 2015-09-24

**Authors:** Sun Jae Jung, Sue Kyung Park, Aesun Shin, Sang-Ah Lee, Ji-Yeob Choi, Yun-Chul Hong, Keun-Young Yoo, Jong-Koo Lee, Daehee Kang

**Affiliations:** Department of Biomedical Science, Seoul National University College of Medicine, Seoul, South Korea; Department of Preventive Medicine, Seoul National University College of Medicine, Seoul, South Korea; Cancer Research Institute, Seoul National University Hospital, Seoul, South Korea; Department of Preventive Medicine, Kangwon National University, Seoul, South Korea; Department of Family Medicine, Seoul National University Hospital, Seoul, South Korea

## Abstract

**Background:**

Spontaneous abortion (SA) affects 11.2% of recognized pregnancies in Korea. Many studies have focused on the increased risk of SA in obese populations, but there are few studies that have focused on underweight (Body mass index (BMI) <18.5 kg/m2) women, especially in relation to pre-pregnancy BMI. The aim of this study was to examine the role of pre-pregnancy BMI at age 18–20 in later SA.

**Methods:**

Among the women who were ever pregnant in the Health Examinees Study (HEXA), which was one of the cohorts studied in the KoGES (Korean Genome and Epidemiology Study) from 2004 to 2012 (*N* = 80,447), the likelihood of SA based on pre-pregnancy BMI, classified by the criteria for Asians (Underweight: <18.5 kg/m2; Normal range: 18.5-22.9 kg/m2; Overweight at risk: 23–24.9 kg/m2; Obese I: 25–29.9 kg/m2; Obese II: ≥30 kg/m2), was presented as odds ratios (ORs) and 95% confidence intervals (95% CIs) using logistic regression models.

**Results:**

Being underweight or obese relative to the normal weight range was associated with a higher likelihood of SA (OR = 1.10 [95% CI = 1.05–1.15] in underweight women and OR = 1.06 [95% CI = 0.96–1.16] in obese women), and this effect was much greater in women who experienced recurrent SAs (for three or more SAs: OR = 1.29 [95% CI 1.14–1.46] in underweight women and OR = 1.39 [95% CI 1.09–1.78] in obese women). Obesity was associated with an increased likelihood of SA at a younger age (≤25 years), whereas underweight was associated with an increased OR of SA at an older age (≥26 years).

**Discussion:**

As this study was conducted with baseline data of original cohort which focused on other chronic diseases, recall for previous pregnancy-related information might be less accurate. However, this study shows strength in its large size and prospective potential.

**Conclusions:**

Pre-pregnancy BMI at ages 18–20 years revealed a U-shaped association with SA, and underweight and obese women showed increased likelihood for SA during different age periods.

**Electronic supplementary material:**

The online version of this article (doi:10.1186/s12884-015-0665-2) contains supplementary material, which is available to authorized users.

## Background

Spontaneous abortion (SA), or miscarriage, is defined as a clinically observed pregnancy that is lost or that ends before the 20th week of gestation [1]. According to the Korean national survey on Fertility, Family Health & Welfare 2012, SA affects 11.2% of recognized pregnancies, which can be viewed as a rather frequent medical phenomenon [2].

Maternal overweight and obesity are related to increased risk of stillbirth, neonatal and infant death, large for gestational age (LGA), fetal malformations, maternal diabetes, pregnancy-induced hypertension, pre-eclampsia and a high frequency of Caesarean section [3–6]. In obese women, folliculogenesis, ovulation and conception are more likely to be impaired, and pregnancy complications frequently occur [7].

In accordance with the known mechanism linking obesity and SA, there are multiple epidemiologic studies reporting the association between these two factors [8, 9]. Many studies focusing on obese or overweight mothers and their adverse outcomes related to pregnancy have been published [4–6], but studies on underweight mothers are rare [10]. Likewise, an association between pre-pregnancy body mass index (BMI) and adverse pregnancy outcomes has been assessed in several studies [11, 12], but similar studies are uncommon in Asian populations.

It is known that bodyweight in early life is associated with morbidity in adult life [13]. In the Nurses’ Health Study (NHS), women with a BMI greater than 25 kg/m2 at age 18 had an increased risk of premature death, and this association was only partially explained by adult obesity [14]. Likewise, there are studies suggesting a crucial effect of early life BMI on later parity [15, 16]. Another study from NHS with 17 years of follow-up also revealed that BMI at age 18 was associated with anovulatory infertility [17]. As BMI in the early years of life seems to affect maternal health, in this study, we aim to evaluate the association between underweight, as well as obesity, at age 18–20 and the risk of SA in the Korean population.

## Methods

### Subject selection

The eligible population was selected from the Health Examinees Study (HEXA). The HEXA comprises a large-scale genomic community-based prospective cohort of 170,094 participants and biological samples between 2004 and 2012. The HEXA was specifically conducted to evaluate epidemiologic characteristics and genomic risk factors focusing on major chronic diseases, including cancer, in the Korean population. It is a component of the Korean Genome and Epidemiology Study (KoGES). Participating centers were chosen after strict evaluation of the selection criteria and community representativeness of participating hospitals. Recruited subjects were drawn from the community’s adult population, ages 40 to 69 years. Information on general lifestyle, including smoking and drinking habits, physical activity, past medical history, family medical history, drug use (including health supplements), health check-ups, psychological factors, diet and reproductive factors, was obtained by trained interviewers with a structured questionnaire. This questionnaire was developed from an extensive literature review of other questionnaires, including the Korea National Health and Nutrition Examination Survey (KNHANES) [18], which is a representative epidemiologic study of Korea funded by the Korean Center for Disease Control (KCDC). The questionnaire was validated using back translation with English, and a pilot study was conducted to evaluate its efficacy and feasibility [19]. Furthermore, anthropometric measures were performed by trained interviewers, and laboratory tests for blood and urine were conducted by central laboratory. More details about the HEXA can be found elsewhere [19].

Of the total participants, 111,600 participants were women. Among them, 8,605 women were excluded because they had an age other than 40–69 years, no experience of pregnancy, or no information on their parity. Additionally, we excluded the 20,729 women who had missing information on weight or height at ages 18–20 years. A total of 1,244 women with a first pregnancy before age 18 or 20 or with unknown age of first pregnancy and 316 women with missing data on spontaneous abortion were also excluded. Finally, a total of 80,447 women were included in the final analysis.

### Measurements

The participants were asked about their general characteristics, including occupations, lifestyle factors, socio-psychological factors, past medical history, family history for medical diseases, surgical history, list of medications and dietary habits. Additionally, the participants were asked about factors associated with obstetrics and gynecology. Women were also asked whether they had ever had a SA; if the answer was ‘yes’, then they were asked at what age the SA had occurred. Additionally, participants were asked about other factors, such as age at menarche, age at first pregnancy, gestational hypertension and gestational diabetes.

Women were asked to recall their weight at age 20 years (surveys from 2004 to 2006) or at age 18 years (surveys from 2007 to 2012). From 2004 to 2006, women had to report their weight at 20 years, but a corresponding question changed to asking the weight at age 18 instead. Therefore, women who participated in the surveys between 2007 and 2012 reported their recalled weight at age 18 only. BMI at age 18 or 20 years was calculated using the measured height at the baseline and dividing the weight by the height squared (kg/m2).

### Classification of BMI

We modified the BMI criteria of the Steering Committee of the Regional Office for the Western Pacific Region of the World Health Organization (WHO-WPRO), which the International Association for the Study of Obesity and the International Obesity Task Force had suggested to be the proper categorization of obesity in Asians. The criteria defines ‘underweight’, ‘normal range’, ‘overweight at risk’, ‘obese I’, and ‘obese II’ as a BMI under 18.5 kg/m2, between 18.5–22.9 kg/m2, between 23–24.9 kg/m2, between 25–29.9 kg/m2, and 30 kg/m2 or higher, respectively [20]. According to these criteria, people who were either ‘obese I’ or ‘obese II’ comprised only 3% of the study population. For this reason, women who were in either the ‘obese I’ or ‘obese II’ group were placed into one group (‘obese’). For the reference group, the ‘normal range’ group was selected.

### Statistical analysis

Multivariable logistic regression was applied to compute odds ratios (ORs) and 95% confidence intervals (95% CI) of the association between BMI at ages 18–20 years and SA. Possible confounders, such as participants’ age at the survey, income, education, comorbid diseases, exercise, cigarette smoking, alcohol drinking, gestational hypertension and gestational diabetes, were selected based on a literature review. Covariates that altered the odds ratio (OR) for at least one SA in the underweight population by 10% compared to the original model were selected.

Initially, the outcome variable was SA; then, we further defined multiple SAs as variables for those who had more than one SA. First, we analyzed the association between pre-pregnancy BMI and women who ever had an SA. Consequently, women who experienced a SA more than twice were classified as the ‘event group’, compared to those who never had a SA or experienced a SA only once. Similarly, those who had a SA at least three times were defined as the ‘event group’ in a later analysis. Additionally, we classified these cases by age at first SA to evaluate the effects of BMI across distinct age groups. Women who experienced SA were divided into 3 groups (≤25 years, 26–28 years, and >28 years) by tertile distribution of age at first SA. Additionally, as both BMI and spontaneous abortion showed associations with gestational diabetes [21–23] and gestational hypertension [22, 24], we performed a stratification analysis on the association between BMI and spontaneous abortions according to these two comorbidities to assess a potential effect modifications.

For statistical analysis, SAS version 9.3 (SAS Institute Inc., Cary, NC, USA) was used. We set P-values less than 0.05 to indicate significance.

### Ethics

Written consent was provided by all subjects who agreed to participate in the HEXA. The statistical analysis of publicly available data was approved by the Institutional Review Board of the Seoul National University Hospital in Seoul, Korea (IRB No.1408-087-604).

## Results

Women who experienced SA were more likely to have more than a high-school education compared to women without an SA experience (25.2% vs. 23.3%) and had an older age at first pregnancy (≥27 years) than women without SA experience (31.9% vs. 29.3%). The proportion of women who had gestational diabetes (GDM) or gestational hypertension (GHT) also differed between SA and non-SA groups (GDM: 1.18% with SA vs. 0.91% without SA; GHT: 6.0% with SA vs. 4.7% without SA). The proportion of women with babies over 4.0 kg (macrosomia) or under 2.5 kg (microsomia) also differed between the two groups (macrosomia: 8.2% with SA vs. 7.0% without SA; microsomia: 5.6% with SA vs. 3.8% without SA) (Table 1).

**Table 1:** General characteristics of the women population with spontaneous abortion (SA) and non-SA at the time of enrollment in the Health Examinee Study (HEXA), a cohort study of the Korean Genome and Epidemiology Study (KoGES) in the Korea Center for Disease Control and Prevention (KCDC), 2004-2012

No.=80,447	Spontaneous Abortion		
	No	Yes	
	*N*=62,637	*N*=17,810	
	N (%)	N (%)	*p*-value*
Attained education ≥12 years	14,598 (23.3)	4,484 (25.2)	<0.001
Cigarette smoking before the first pregnancy	538 (0.859)	154 (0.865)	0.002
Ever consumed alcohol^1^	21,222 (33.9)	6,139 (34.5)	<0.001
Regular exercise	32,437 (51.8)	9,211 (51.7)	0.305
Medical history before pregnancy			
Hypertension	37 (0.06)	9 (0.05)	0.687
Diabetes	20 (0.02)	3 (0.02)	0.291
Chronic liver disease	154 (0.25)	35 (0.20)	0.266
Tuberculosis	2,067 (3.30)	587 (3.30)	0.878
Thyroid disease	211 (0.34)	73 (0.41)	0.138
Reproductive factors			
Menarche age (years)			0.071
≤14	23,559 (37.6)	6,830 (38.4)	
15	14,077 (22.5)	3,845 (21.6)	
≥16	23,903 (38.2)	6 816 (38.3)	
First pregnancy age			<0.001
≤23	19,199 (30.7)	5,093 (28.6)	
24–26	25,066 (40.0)	7,029 (39.5)	
≥27	18,372 (29.3)	5,688 (31.9)	
First pregnancy outcome			
Stillbirth	393 (0.63)	178 (1.00)	<0.001
Extrauterine pregnancy	195 (0.31)	67 (0.38)	0.184
Artificial abortion	7,467 (12.0)	1,466 (8.3)	
Number of children			<0.001
1	6,358 (10.2)	2,062 (11.8)	
2	37,754 (60.8)	9,974 (57.0)	
3+	17,866 (28.8)	5,420 (30.1)	
Gestational diabetes	569 (0.91)	211 (1.18)	<0.001
Gestational hypertension	2,966 (4.7)	1,065 (6.0)	<0.001
Macrosomia (>4.0kg)	4,406 (7.0)	1,646 (8.2)	<0.001
Microsomia (<2.5kg)	2,390 (3.8)	995 (5.6)	<0.001

*p–value from *χ* ^2^–test

^1^Due to lack of information about age of beginning of drinking, usual drinkers were included in this category

In the adjusted logistic regression model, a significantly increased likelihood was observed in women who had a BMI lower than 18.5 kg/m2 (OR 1.10, 95% CI 1.05–1.15); conversely, a significantly reduced likelihood of SA was observed in women who had a BMI between 23–24.9 kg/m2 (OR 0.94, 95% CI 0.88–0.99) compared to the reference group with a BMI between 18.5–22.9 kg/m2. The association between BMI and total SAs showed a U-shape pattern when considering all three SA categories. When we defined multiple SA as women who had at least 3 SA, the likelihood significantly increased in both those who had a BMI less than 18.5 kg/m2 and those who had a BMI of 25 kg/m2 or higher at ages 18–20 years (OR 1.29, 95% CI 1.14–1.46 and OR 1.39, 95% CI 1.09–1.78, respectively, for three or more SAs) (Table 2).

**Table 2:** Logistic regression for having spontaneous abortions (SAs) at age 18–20 from the Health Examinees Study (HEXA), 2004–2012

BMI (kg/m^2^)	Total women	Total women with a history of at least			Comparison by groups		
	(*N*=80,447)	One abortion	Two abortion	Three abortion	≥1 SAs vs. No. SA	≥2 SAs vs. 0–1 SA	≥3 SAs vs. 0–2 SA
		(*N*=17,180)	(*N*=4,875)	(*N*=1,704)			
	N	N (%)	N (%)	N (%)	OR^1^ (95% CI)	OR^1^ (95% CI)	OR^1^ (95% CI)
<18.5	13128	3,122 (17.5)	896 (18.38)	335 (19.7)	1.10 (1.05–1.15)	1.14 (1.06–1.23)	1.29 (1.14–1.46)
18.5–22.9	56692	12,462 (70.0)	3,380 (69.3)	1,149 (67.4)	1.00 (ref)	1.00 (ref)	1.00 (ref)
23–24.9	8148	1,675 (9.4)	438 (9.0)	148 (8.7)	0.93 (0.88–0.99)	0.91 (0.82–1.01)	0.88 (0.74–1.04)
≥25	2479	551 (3.1)	161 (3.3)	72 (4.2)	1.06 (0.96–1.16)	1.13 (0.96–1.33)	1.39 (1.09–1.78)

^1^Adjusted for age, education, smoking before first pregnancy, drinking status and first pregnancy age

Different patterns were observed in groups with different ages of onset of SA. In women who had their first SA at the age of 25 years or younger, only those with a BMI of 25 kg/m2 or higher at ages 18–20 years showed a significantly increased likelihood (OR 1.22, 95% CI 1.02–1.45) of SA. In contrast, the likelihood of SA at ages 26 to 28 years decreased in women with a BMI of 25 kg/m2 or higher (OR 0.82, 95% CI 0.66–1.01). In regard to the likelihood of an SA after the age of 28 years, only women with a BMI less than 18.5 kg/m2 at ages 18–20 years showed a marginally significant increased likelihood (OR 1.11, 95% CI 1.02–1.21) (Table 3).

**Table 3:** Stratification analyses by maternal age for the likelihood for total and recurrent spontaneous abortion (SA) of body mass index (BMI) at 18–20 years old in the Health Examinee Study (HEXA), 2004-2012^1^

BMI (kg/m^2^)	Maternal age					
	SA at ≤25 years		SA at 26–28 years		SA at >28 years	
	(*N*=3,980)		(*N*=3,977)		(*N*=4,321)	
	N (%)	OR^2^ (95% CI)	N (%)	OR^2^ (95% CI)	N (%)	OR^2^ (95% CI)
<18.5	636 (16.0)	1.07 (0.97–1.17)	768 (19.3)	1.17 (1.08–1.27)	844 (19.5)	1.11 (1.02–1.21)
18.5–22.9	2,765 (69.5)	1.00 (ref)	2,761 (69.4)	1.00 (ref)	2,989 (69.2)	1.00 (ref)
23–24.9	427 (10.7)	1.02 (0.91–1.32)	356 (8.95)	0.92 (0.82–1.03)	376 (8.7)	0.93 (0.83–1.04)
≥25	153 (3.8)	1.22 (1.02–1.45)	92 (2.3)	0.82 (0.66–1.01)	112 (2.6)	0.96 (0.79–1.17)

^1^Only included participants who were enrolled between 2007 and 2012 due to lack of information in age at spontaneous abortion

^2^Adjusted for age, education, smoking before first pregnancy and drinking status

In the stratification analysis of smoking status before first pregnancy, there were similar patterns in the likelihood of SA according to smoking status with various degrees of ORs, compared to the reference group (BMI 18.5–23 kg/m2). In women who did not smoke before pregnancy, the likelihood significantly increased for those who had a BMI less than 18.5 kg/m2 (OR 1.09, 95% CI 1.05–1.15) and decreased with marginal significance for those who had BMIs between 23–24.9 kg/m2 (OR 0.95, 95% CI 0.89–1.00). In women who smoked before the first pregnancy, those who were ‘underweight’ at ages 18–20 years had an increased odds ratio (OR 1.31, 95% CI 0.70–2.44), whereas the odds ratio in those who had a BMI of 25 kg/m2 or over was decreased (OR 0.74, 95% CI 0.15–3.59), although they were not significant (Table 4).

**Table 4:** Stratification analyses by smoking before pregnancy for the likelihood for total and recurrent spontaneous abortion (SA) by body mass index (BMI) at 18–20 years old in the Health Examinee Study (HEXA), 2004–2012

BMI (kg/m^2^)	Smokers before pregnancy		Never-smokers before pregnancy	
	SA	OR^1^ (95% CI)^1^	SA	OR^1^ (95% CI)^1^
	N (%)		N (%)	
<18.5	34 (26.0)	1.31 (0.70–2.44)	3,060 (17.5)	1.09 (1.05–1.15)
18.5–22.9	49 (67.1)	1.00 (ref)	12,249 (69.9)	1.00 (ref)
23–24.9	3 (4.1)	0.70 (0.12–1.40)	1,666 (9.5)	0.94 (0.89–1.00)
≥25	2 (2.7)	0.74 (0.15–3.59)	545 (3.1)	1.05 (0.95–1.16)

^1^Adjusted by age, education, smoking before pregnancy, drinking status and first pregnancy age

An additional stratification analysis was conducted with gestational diabetes and gestational hypertension. In women with gestational hypertension, only women with BMIs less than 18.5 kg/m2 were associated with a higher likelihood of SA (OR 1.22, 95% CI 1.01–1.49). In those with gestational diabetes, no significantly increased OR was observed in the groups, compared with the reference group. Among women with no gestational diabetes and gestational hypertension, both underweight and obesity were associated with a higher likelihood of SA, and women who were in the overweight range showed significantly decreased likelihoods of SA, which were similar to the patterns observed for the entire population (Additional file 1: Table S1 and Additional file 2: Table S2).

## Discussion

The goal of this study was to evaluate the effect of pre-pregnancy BMI on SA as an independent risk factor in a large Korean population. In the analysis of approximately 80,000 women, both high and low BMIs showed increased likelihoods of SA, resulting in a U-shaped association. In contrast to the expected result, being overweight (BMI 23–24.9 kg/m2) showed the lowest OR of SA. Our results also showed a similar pattern to the results of a study conducted on more than 1 million Asians, in which a harmful effect due to being underweight and a positive effect due to being overweight (22.6–25.0 kg/ m2) on mortality was suggested [25].

Considering the age at the first SA, it appears that a high BMI or being ‘obese’ at ages 18–20 years seems to affect SA at an earlier age, such as 25 years or younger. In contrast, lower BMI at ages 18–20 years or being ‘underweight’ influenced SA at a later age (i.e., more than 25 years). If women had gestational hypertension, those with a BMI under 18.5 kg/m2 tended to have more SAs.

As many studies have focused only on the harm of being obese before pregnancy, the possible association between underweight and reproduction has not received much attention. However, our results suggest that underweight women at pre-pregnancy ages are at higher risk for SA compared to normal weight women.

According to the literature review, there is evidence that an increase in BMI raises the risk of SA in the general population [26], as well as in women who have undergone infertility treatment [8]. However, the relationship between lower BMI and SA is far from conclusive, especially in Asians. In a Danish National Birth Cohort (DNBC), 23,000 women were analyzed, and women with a pre-pregnancy BMI under 18.5 kg/m2 or higher than 25 kg/m2 had an increased hazard ratio (HR) of SA (HR 1.24, 95% CI 0.95–1.63; HR 1.14 95% CI 0.98–1.31, respectively) [10]. In comparison, our results show significantly increased ORs in both ‘underweight’ and ‘obese’ populations, demonstrating a U-shaped pattern.

In regard to the association between BMI and multiple recurrent SA, women with a higher BMI were considered to have a greater likelihood of SA. A study conducted in the Guangzhou Chinese population reported significantly increased likelihoods of recurrent SA in participants with a BMI of 24 kg/m2 or higher (OR 1.54; 95% CI 1.28–3.49) compared to their counterparts [27]. Because this study only compared those with a BMI of 24 kg/m2 or higher to those with a BMI under 24 kg/m2, it was impossible to evaluate the effect of lower BMIs on SA. Another study conducted in the U.K. analyzed 844 pregnancies of 491 women with recurrent SA. In this study, women with a BMI under 19 kg/m2 or over 30 kg/m2 had increased ORs, but neither group was significant [28].

Given the number of studies regarding the effect of smoking on SA, studies comparing the effect of BMI on both smokers and non-smokers are scarce. Most studies evaluate the effect of smoking and BMI on SA separately [29]. A study conducted in Sweden reported that the interaction among age, BMI and smoking was not statistically significant [29]. However, as seen in our results, the likelihood of SA increases in both ‘underweight’ and ‘obese’ women, and this association is not linear. Additionally, it is well known that as BMI increases, the risk of hypertensive disorders also increases [30]. Interestingly, in women who had gestational hypertension, we observed significantly increased ORs only in the ‘underweight’ population.

There is abundant evidence supporting the biological plausibility of the extended risk of higher BMIs and the risk of SA. Adolescent or early adult obesity is related to increased overall mortality and especially and an increased risk of diabetes and cardiovascular diseases in adult life [31]. The association between obesity in youth and inflammation leads to vascular damage over time. Chronic inflammation may have a key role in SA by damaging the vessels, which support the utero-feto-placental complex [32].

In contrast, there are also studies supporting the mechanism of SA in low BMI women. It has been reported that women with low weights have an increased risk of fetal growth retardation [33], which can be explained by an increased rate of vasoconstriction and decreased serum glucose levels due to poor diet and decreased blood pressure, provoking reduced placental perfusion [34]. Additionally, it has been proposed that leptin, a hormone secreted by adipose cells, may play a role in this association. Underweight women usually have low levels of this hormone, which can block ovulation [35]. Similarly, maternal underweight caused by a shortage of food or eating disorders, such as anorexia or bulimia, is associated with SA [36, 37].

There are several limitations to this study. First, as the original HEXA was designed to mainly focus on other chronic diseases, the recall of retrospective, pregnancy-related information might be less accurate. However, as participants were not aware of the main purpose of this study, there is less chance for recall bias. Additionally, the mean age at enrollment was 52.0 years, and the women were asked to recall their weight nearly 30 years prior. The results of the Nurses’ Health Study (NHS) indicate that participants were able to recall their weight at the age of 18 years, and the correlation coefficient between recalled and objective measurements for weight was approximately 0.9 [38]. Although women in the NHS were aged 25 to 42 years were therefore younger than our participants, there is evidence that self-reported height and weight data are adequate for assessing associations in epidemiological studies [39]. In the stratification analysis of cigarette smoking before pregnancy, we had an insufficient number of samples to explore the effect of the association of first age of smoking and BMI at ages 18–20 years and SA.

This study’s strength is its large size and prospective potential. To the best of our knowledge, it is currently the largest study that has addressed pre-pregnancy BMI and SA. Additionally, although this study used baseline cohort data with cross-sectional analysis, temporal characteristics are included in the analysis, as women with BMIs at ages 18–20 years were analyzed. Reverse causation is the most important problem, in which the loss of weight resulting from particular diseases can falsify the association between lower BMI and health outcomes. To clarify this problem, we excluded women who had pregnancies before the ages of 18–20 years. Furthermore, to provide reliable estimated values for the total effect of BMI on the likelihood of SA in a large Korean population, this study also evaluated the relationship between low BMI and the likelihood of SA in a more discrete way. This particular outcome could not be appropriately demonstrated in most of the earlier studies conducted in European populations.

## Conclusions

This study revealed that pre-pregnancy BMI at ages 18–20 years was associated with a higher likelihood of SA in underweight or obese women, using the Asian BMI classification. This U-shaped association was much stronger for recurrent, consequent SA or in women who had ever smoked cigarettes before pregnancy. Age at SA and gestational hypertension modified the association of pre-pregnancy BMI with SA. Obese women had a higher likelihood of SA at earlier maternal ages, whereas underweight women had a higher likelihood of SA at typical or later ages or with gestational hypertension. Further studies are needed to evaluate the exact cause of the difference in age trends and BMI ranges.

## Additional files

Additional file 1: Table S1. Stratification analyses by gestational diabetes (GDM) for the likelihood for total and recurrent spontaneous abortion (SA) of body mass index (BMI) at 18–20 years old in the Health Examinee Study (HEXA), 2004–2012. (DOC 33 kb) Additional file 2: Table S2. Stratification analyses by gestational hypertension (GHT) for the likelihood for total and recurrent spontaneous abortion (SA) of body mass index (BMI) at 18–20 years old in the Health Examinee Study (HEXA), 2004–2012. (DOC 33 kb)

## References

[1] Regan L, Rai R (2000). Epidemiology and the medical causes of miscarriage. Baillieres Best Pract Res Clin Obstet Gynaecol.

[2] Kim SG, Kim YK, Kim HR, Park JS, Sohn CK, Choi YJ (2012). The 2012 national survey on fertility, family health & welfare in Korea.

[3] Yazdani S, Yosofniyapasha Y, Nasab BH, Mojaveri MH, Bouzari Z (2012). Effect of maternal body mass index on pregnancy outcome and newborn weight. BMC research notes.

[4] Cnattingius S, Bergstrom R, Lipworth L, Kramer MS (1998). Prepregnancy weight and the risk of adverse pregnancy outcomes. N Engl J Med.

[5] Ehrenthal DB, Jurkovitz C, Hoffman M, Jiang X, Weintraub WS (2011). Prepregnancy body mass index as an independent risk factor for pregnancy-induced hypertension. J Womens Health (Larchmt).

[6] Dennedy MC, Avalos G, O’Reilly MW, O’Sullivan EP, Gaffney G, Dunne F (2012). ATLANTIC-DIP: raised maternal body mass index (BMI) adversely affects maternal and fetal outcomes in glucose-tolerant women according to International Association of Diabetes and Pregnancy Study Groups (IADPSG) criteria. J Clin Endocrinol Metab.

[7] Athukorala C, Rumbold AR, Willson KJ, Crowther CA (2010). The risk of adverse pregnancy outcomes in women who are overweight or obese. BMC Pregnancy Childbirth.

[8] Wang JX, Davies MJ, Norman RJ (2002). Obesity increases the risk of spontaneous abortion during infertility treatment. Obes Res.

[9] Boots C, Stephenson MD (2011). Does obesity increase the risk of miscarriage in spontaneous conception: a systematic review. Semin Reprod Med.

[10] Helgstrand S, Andersen AM (2005). Maternal underweight and the risk of spontaneous abortion. Acta Obstet Gynecol Scand.

[11] Bhattacharya S, Campbell DM, Liston WA, Bhattacharya S (2007). Effect of body mass index on pregnancy outcomes in nulliparous women delivering singleton babies. BMC Public Health.

[12] Dzakpasu S, Fahey J, Kirby RS, Tough SC, Chalmers B, Heaman MI (2015). Contribution of prepregnancy body mass index and gestational weight gain to adverse neonatal outcomes: population attributable fractions for Canada. BMC Pregnancy Childbirth.

[13] Strauss RS, Pollack HA (2001). Epidemic increase in childhood overweight, 1986–1998. JAMA.

[14] van Dam RM, Willett WC, Manson JE, Hu FB (2006). The relationship between overweight in adolescence and premature death in women. Ann Intern Med.

[15] Polotsky AJ, Hailpern SM, Skurnick JH, Lo JC, Sternfeld B, Santoro N (2010). Association of adolescent obesity and lifetime nulliparity--the Study of Women’s Health Across the Nation (SWAN). Fertil Steril.

[16] Sukalich S, Mingione MJ, Glantz JC (2006). Obstetric outcomes in overweight and obese adolescents. Am J Obstet Gynecol.

[17] Rich-Edwards JW, Goldman MB, Willett WC, Hunter DJ, Stampfer MJ, Colditz GA (1994). Adolescent body mass index and infertility caused by ovulatory disorder. Am J Obstet Gynecol.

[18] Kweon S, Kim Y, Jang MJ, Kim Y, Kim K, Choi S (2014). Data resource profile: the Korea National Health and Nutrition Examination Survey (KNHANES). Int J Epidemiol.

[19] Kang D, Lee J.K, Kim SS, Han B G. The Health Examinees (HEXA) study: rationale, study design, and baseline characteristics. Asian Pac J Cancer Prev. 2015, 15(In press). 10.7314/apjcp.2015.16.4.1591 25743837

[20] Anuurad E, Shiwaku K, Nogi A, Kitajima K, Enkhmaa B, Shimono K (2003). The new BMI criteria for asians by the regional office for the western pacific region of WHO are suitable for screening of overweight to prevent metabolic syndrome in elder Japanese workers. J Occup Health.

[21] Ogonowski J, Miazgowski T, Kuczynska M, Krzyzanowska-Swiniarska B, Celewicz Z (2009). Pregravid body mass index as a predictor of gestational diabetes mellitus. Diabetic medicine : a journal of the British Diabetic Association.

[22] Shin D, Song WO. Prepregnancy body mass index is an independent risk factor for gestational hypertension, gestational diabetes, preterm labor, and small-and large-for-gestational-age infants. J Matern Neonatal Med. 2014;1–8. 10.3109/14767058.2014.964675 25211384

[23] Saydah SH, Chandra A, Eberhardt MS (2005). Pregnancy experience among women with and without gestational diabetes in the U.S., 1995 National Survey Of Family Growth. Diabetes Care.

[24] Silverstone A, Trudinger BJ, Lewis PJ, Bulpitt CJ (1980). Maternal hypertension and intrauterine fetal death in mid-pregnancy. Br J Obstet Gynaecol.

[25] Zheng W, McLerran DF, Rolland B, Zhang X, Inoue M, Matsuo K (2011). Association between body-mass index and risk of death in more than 1 million Asians. N Engl J Med.

[26] Metwally M, Ong KJ, Ledger WL, Li TC (2008). Does high body mass index increase the risk of miscarriage after spontaneous and assisted conception? A meta-analysis of the evidence. Fertil Steril.

[27] Zhang BY, Wei YS, Niu JM, Li Y, Miao ZL, Wang ZN (2010). Risk factors for unexplained recurrent spontaneous abortion in a population from southern China. Int J Gynaecol Obstet.

[28] Metwally M, Saravelos SH, Ledger WL, Li TC (2010). Body mass index and risk of miscarriage in women with recurrent miscarriage. Fertil Steril.

[29] Waldenstrom U, Aasheim V, Nilsen AB, Rasmussen S, Pettersson HJ, Schytt E (2014). Adverse pregnancy outcomes related to advanced maternal age compared with smoking and being overweight. Obstet Gynecol.

[30] Dantas EM, Pereira FV, Queiroz JW, Dantas DL, Monteiro GR, Duggal P (2013). Preeclampsia is associated with increased maternal body weight in a northeastern Brazilian population. BMC Pregnancy Childbirth.

[31] Lappas M, Permezel M, Rice GE (2005). Leptin and adiponectin stimulate the release of proinflammatory cytokines and prostaglandins from human placenta and maternal adipose tissue via nuclear factor-kappaB, peroxisomal proliferator-activated receptor-gamma and extracellularly regulated kinase 1/2. Endocrinology.

[32] Kohut KG, Anthony MN, Salafia CM (1997). Decidual and placental histologic findings in patients experiencing spontaneous abortions in relation to pregnancy order. Am J Reprod Immunol.

[33] Isaksen CV, Laurini RN, Jacobsen G (1997). Pre-pregnancy risk factors of small-for-gestational-age births and perinatal mortality. Acta Obstet Gynecol Scand Suppl.

[34] Cliver SP, Goldenberg RL, Cutter GR, Hoffman HJ, Copper RL, Gotlieb SJ (1992). The relationships among psychosocial profile, maternal size, and smoking in predicting fetal growth retardation. Obstet Gynecol.

[35] Bolumar F, Olsen J, Rebagliato M, Saez-Lloret I, Bisanti L (2000). Body mass index and delayed conception: a European Multicenter Study on Infertility and Subfecundity. Am J Epidemiol.

[36] Franko DL, Walton BE (1993). Pregnancy and eating disorders: a review and clinical implications. Int J Eat Disord.

[37] Wynn A, Wynn M (1993). The effects of food shortage on human reproduction. Nutr Health.

[38] Troy LM, Hunter DJ, Manson JE, Colditz GA, Stampfer MJ, Willett WC (1995). The validity of recalled weight among younger women. International journal of obesity and related metabolic disorders : journal of the International Association for the Study of Obesity.

[39] Strauss RS (1999). Comparison of measured and self-reported weight and height in a cross-sectional sample of young adolescents. Int J Obes Relat Metab Disord.

