# Supplementary material for: Body mass index at age 18–20 and later risk of spontaneous abortion in the Health Examinees Study (HEXA)
Source: BMC Pregnancy Childbirth. 2015 Sep 24;15:228. doi: 10.1186/s12884-015-0665-2 (PMC4582827; doi:10.1186/s12884-015-0665-2)
Supplement: Additional file 1: Table S1. — Stratification analyses by gestational diabetes (GDM) for the likelihood for total and recurrent spontaneous abortion (SA) of body mass index (BMI) at 18–20 years old in the Health Examinee Study (HEXA), 2004–2012. (DOC 33 kb) [file 12884_2015_665_MOESM1_ESM.doc]

# Additional files

### Supplementary 1. Stratification analyses by gestational diabetes (GDM) for the likelihood for total and recurrent spontaneous abortion (SA) of body mass index (BMI) at 18-20 years old in the Health Examinee Study (HEXA), 2004-2012

| BMI (kg/m^2^) |  | With gestational diabetes | | |  | Without gestational diabetes | | |
| --- | --- | --- | --- | --- | --- | --- | --- | --- |
|  |  | SA | No SA | OR^1^ (95% CI)^1^ |  | SA | No SA | OR^1^ (95% CI)^1^ |
|  |  | N (%) | N(%) |  |  | N (%) | N(%) |  |
| <18.5 |  | 39 (18.5) | 92 (16.2) | 1.04 (0.68-1.59) |  | 2,918 (17.5) | 9,498 (16.0) | **1.09 (1.04-1.14)** |
| 18.5-22.9 |  | 154 (73.0) | 389 (68.4) | 1.00 (ref) |  | 11,627 (69.9) | 41,874 (70.6) | 1.00 (ref) |
| 23-24.9 |  | 16 (7.6) | 64 (11.3) | 0.65 (0.36-1.16) |  | 1,578 (9.5) | 3,162 (10.3) | **0.94 (0.89-1.00)** |
| ≥25 |  | 2 (1.0) | 24 (4.2) | 0.19 (0.04-0.82) |  | 522 (3.1) | 1,825 (3.1) | 1.06 (0.96-1.17) |
| 1. Adjusted by age, education, smoking before pregnancy, drinking status and first pregnancy age | | | | | | | | |
